# Characterization of *in vitro* haploid and doubled haploid *Chrysanthemum morifolium* plants via unfertilized ovule culture for phenotypical traits and DNA methylation pattern

**DOI:** 10.3389/fpls.2014.00738

**Published:** 2014-12-22

**Authors:** Haibin Wang, Bin Dong, Jiafu Jiang, Weimin Fang, Zhiyong Guan, Yuan Liao, Sumei Chen, Fadi Chen

**Affiliations:** ^1^College of Horticulture, Nanjing Agricultural UniversityNanjing, China; ^2^Jiangsu Province Engineering Lab for Modern Facility Agriculture Technology & EquipmentNanjing, China

**Keywords:** chrysanthemum, haploid, double haploid, ovule culture, MSAP

## Abstract

Chrysanthemum is one of important ornamental species in the world. Its highly heterozygous state complicates molecular analysis, so it is of interest to derive haploid forms. A total of 2579 non-fertilized chrysanthemum ovules pollinated by *Argyranthemum frutescens* were cultured *in vitro* to isolate haploid progeny. One single regenerant emerged from each of three of the 105 calli produced. Chromosome counts and microsatellite fingerprinting showed that only one of the regenerants was a true haploid. Nine doubled haploid derivatives were subsequently generated by colchicine treatment of 80 *in vitro* cultured haploid nodal segments. Morphological screening showed that the haploid plant was shorter than the doubled haploids, and developed smaller leaves, flowers, and stomata. An *in vitro* pollen germination test showed that few of the haploid's pollen were able to germinate and those which did so were abnormal. Both the haploid and the doubled haploids produced yellow flowers, whereas those of the maternal parental cultivar were mauve. Methylation-sensitive amplification polymorphism (MSAP) profiling was further used to detect alterations in cytosine methylation caused by the haploidization and/or the chromosome doubling processes. While 52.2% of the resulting amplified fragments were cytosine methylated in the maternal parent's genome, the corresponding proportions for the haploid's and doubled haploids' genomes were, respectively, 47.0 and 51.7%, demonstrating a reduction in global cytosine methylation caused by haploidization and a partial recovery following chromosome doubling.

## Introduction

Chrysanthemum (*Chrysanthemum morifolium*) is a leading ornamental species and enjoys a wide acceptance in the world (Hall and Dickson, 2011; Teixeira da Silva et al., 2013). Intensive breeding has produced a large array of flower color and form, Nevertheless, market pressure for further innovation has driven the industry to continue to seek novelty, along with a continuous need to improve levels of biotic and abiotic stress resistance (Chandler and Sanchez, 2012). The allohexaploid status of the chrysanthemum's genome complicates breeding advance, which has become a major bottleneck for *C. morifolium* breeding, especially in the context of applying molecular breeding technologies (Kearsey, 2002; Thomas et al., 2003; Teixeira da Silva et al., 2013).

During the last decade, the current DNA sequencing platforms have had a major impact on plant genetics and breeding technology (Loman et al., 2012). However, species such as *C. morifolium*, which possess large and highly heterozygous genomes, remain difficult to handle at the sequence level (Shendure and Ji, 2008; Loman et al., 2012; Wang et al., 2014a,b). Fortunately, a major simplification can be achieved if haploid plants can be raised from these species (Germanà, 2011; Li et al., 2013a). Since the successful haploidization of *Datura stramonium* (Blakeslee et al., 1922), a number of *in vitro* techniques have been developed to allow the successful generation of haploids from a wide range of species (Maluszynski, 2003; Clarke et al., 2011).

A common haploidization approach is to seek to regenerate plants from non-fertilized female (gynogenesis) or male (androgenesis) gametes by culturing the appropriate explant *in vitro* (Chen et al., 2011; Clarke et al., 2011; Germanà, 2011). Gynogenesis has proven to be a successful method in a number of species (Muren, 1989; Hansen et al., 1995; Alan et al., 2003; Godbole and Murthy, 2012). In addition to their value for genomic analysis, haploids can also make an important contribution to crop improvement, since they can be used to short-circuit the process of gene fixation which otherwise requires a number of generations of self fertilization (Godbole and Murthy, 2012; Mohammadi et al., 2012). In self-incompatible species such as chrysanthemum, however, achieving fixation via self-fertilization is scarcely possible.

Methylation of the cytosine base [especially methylation of cytosine at position 5 (5-methylcytosine, 5^m^C)] has been recognized as the predominant form of epigenetic modification, and thus is an important determinant of the information content of eukaryotic genomes. A number of transcribed sequences are characterized by having a lower level of cytosine methylation than do silent ones, especially in their promoter region (Chan et al., 2005). Variation with respect to patterns of DNA methylation has been widely exploited to make inferences regarding plant evolution (Rapp and Wendel, 2005; Leitch and Leitch, 2008; Koh et al., 2010), and, in particular, to the consequences of allopolyploidization (Chen, 2007; Wang et al., 2013b, 2014a). Here, an attempt has been made to generate haploid chrysanthemum plants by stimulating the ovules prior to their *in vitro* culture through the germination of incompatible pollen on the stigma. Changes to the morphology of the resulting haploids and doubled haploids have been documented, and the methylation-sensitive amplification polymorphism (MSAP) platform used to characterize epigenetic changes induced by both the haploidization and chromosome doubling processes.

## Materials and methods

### Plant materials and mock pollination

The chrysanthemum cultivar “Zhongshanzigui” (2*n* = 6*x* = 54) was pollinated by the diploid species *Argyranthemum frutescens* (2*n* = 2*x* = 18); both “Zhongshanzigui” and the *A. frutescens* accession used are maintained by the Chrysanthemum Germplasm Resource Preserving Centre, Nanjing Agricultural University, China (32°05′N, 118°8′E, 58 m altitude). Bisexual florets were removed from the maternal inflorescence, and the flowers covered with a paper bag at the stage when the stigmas first became visible. Pollination was carried out 2 days later using a fine-haired brush at 10:00–11:00 AM on a sunny day. To track the progress of pollen growth, 10 ligulate flowers were harvested at six, 12, 24, 36, and 48 h after pollination, and fixed at room temperature in 1:1:18 formalin:glacial acetic acid:70% ethanol (v/v). Prior to its microscopic examination, the material was softened by immersion in 1 M NaOH for 12 h, squashed under a cover slip and visualized by a fluorescence microscope (Sun et al., 2010).

### Ovule culture

Two days after pollination, the ovaries were harvested, surface-sterilized by immersion in 75% (v/v) ethanol for 35 s, followed by 10% (v/v) H_2_O_2_ for 15 min and six rinses in sterile water. The ovules were dissected from the ovaries and placed on Murashige and Skoog (MS) (Murashige and Skoog, 1962) medium (pH 5.8) containing 8.88 mM 6-benzyladenine, 2.68 mM α-naphthaleneacetic acid (NAA) (Tang et al., 2009), 3% (w/v) sucrose and solidified with 0.7% (w/v) agar. The explants were held at 25 ± 1°C under a 16 h photoperiod provided by cool white fluorescent lamps (30 μmol m^−2^ s^−1^). Regenerating plantlets were transferred to fresh MS medium (pH 5.7) containing 0.54 mM NAA, 1% (w/v) sucrose and solidified by 0.7% agar, where they were maintained until emerging roots had extended to a length of ~1 cm, at which point they were transplanted into a 2:2:1 mixture of perlite, vermiculite and leaf mold, and maintained in a greenhouse.

### Ploidy level and origin evaluation

Meiotic chromosome pairing in cv. “Zhongshanzigui” was monitored in the anthers of immature inflorescences (6–7 mm diameter) fixed in Carnoy's solution (three parts 95% ethanol, one part glacial acetic acid, v/v). The ploidy level of the regenerants was deduced from their somatic chromosome number, as assayed in their root tips. Roots of ~1 cm in length were held in ice water for 20–24 h, then fixed in Carnoy's solution and stored at 4°C for 24 h. Both anthers and root tips were squashed in a drop of 45% acetic acid and the mitotic chromosomes were visualized using phase contrast microscopy.

The genomic constitution of the regenerants was assessed via microsatellite genotyping. Genomic DNA was isolated from fully expanded 9th and 10th leaves (counting from the apex) using a modified CTAB method (Porebski et al., 1997). The eight informative primer pairs used are detailed in Table 1. Each PCR comprised an initial denaturation of 95°C/5 min, followed by seven cycles of 94°C/45 s, 68°C/45 s (decreasing by 2°C per cycle), 72°C/60 s, then 30 cycles of 94°C/45 s, 54°C/45 s, 72°C/60 s, and finally an extension step of 72°C/5 min. The resulting amplicons were separated by electrophoresis through a 6% (w/v) polyacrylamide gel and visualized by silver staining (Wang et al., 2013a).

**Table 1:** **Sequences of primers used for microsatellite fingerprinting**.

**Primers ID**	**Sequence (5′–3′)**
5-F	AAGAAGAACACCAACGCACC
5-R	CAGAACCTGCACGCATTCTA
7-F	AAACGTGGTTTGCTGAAAGG
7-R	ATTGGGCAAACATCAAAAGC
9-F	AGCCAGGGTGTTGAAAATTG
9-R	ATCAGTCACCCCACTCGAAC
21-F	GTTCGCCGCTAAACAAAAAC
21-R	GGGATTGGATTTCAAGGGAT
34-F	CCTAGTATCAAAGCTGCGAACA
34-R	CAATCGCGTTATCGTGTACC
52-F	AGTGACCCGAGCCAGATAGA
52-R	CCGACAAATCATTTCCGTCT
53-F	TCGAAGACAATCAGCACCTG
53-R	TAAGTGTTCTTCCAGCGCCT
77-F	AGGAGGACAATTCGTGCAAC
77-R	CCGTATACCACCAATACAAATACA

### Chromosome doubling

Doubled haploids were induced by colchicine treatment. A set of 80 nodal segments harvested from a 1 month old *in vitro* cultured haploid plantlet were immersed in 500 mg/L colchicine for 48 h (Liu et al., 2011), then rinsed four times in sterile water and cultured for 30 days on solidified (0.7% agar) MS medium (pH 5.8) containing 3.0% (w/v) sucrose. At the end of this period, the material was transferred to a rooting medium (solidified MS supplemented with 0.54 mM NAA, 1% sucrose, pH 5.7) and maintained for 60 days at 25 ± 1°C under a 16 h photoperiod provided by cool white fluorescent lamps (30 μmol m^−2^ s^−1^). Finally, the plantlets were transplanted into soil and raised in a greenhouse.

### Morphological characterization

Ten individual plants were employed for morphological characterization. Plant height and various flower and leaf traits were recorded. An inflorescence diameter index was calculated, and the number of ligulate florets and tubular florets was averaged from a sample of 10 inflorescences (Li, 1993). Leaf length and width were mesaured for the fifth leaf below the shoot apex and average was calaculated from a sample of 10 plants. The length and width of the stomata were measured using an eyepiece micrometer, again from a sample of 10 leaves. Pollen fertility was determined by monitoring germination by a fluorescence microscope after an 8 h incubation at 20°C on an optimized medium composed of ME_3_, 0.1 g/L H_3_BO_3_, 0.3 g/L CaCl_2_·2H_2_O, and 200 g/L PEG4000 (Li et al., 2009). Mean germination rates were calculated from three replicates of at least 100 pollen grains. The resulting data were statistically analyzed using SPSS v17.0 software (SPSS, Chicago, IL, USA). An analysis of variance was conducted, employing the Tukey test to identify means which differed significantly from one another.

### MSAP analysis

The DNA was subjected to MSAP analysis (Vos et al., 1995; Benhattar and Clement, 2004; Roberts et al., 2007), using as adaptors and primers the sequences shown in Table 2. The selective amplification reactions were based on one of 30 primer combinations (E2+HM3/6/7/8, E3+HM3/8, E4+HM2/3/6/7/8, E5+HM1/2/7/8, E6+HM1/2/6/8, E7+HM1/3/6/7/8, and E8+HM1/2/3/6/7/8, where E indicates *Eco*R I and HM *Hpa* II or *Msp* I). Meanwhile, two randomly selected plants per line were analyzed with a sub-group of the most informative primer combinations (Wang et al., 2013b, 2014a) to determine whether differences in cytosine methylation profile existed between biological replicates. The amplicons were separated by denaturing polyacrylamide gel electrophoresis and detected by silver staining, following Vos et al. (1995). Fragments were scored as present/absent, and only considered if they were reproducible across two independent PCRs. A statistical analysis of differences between the parent and each of the derivatives (*U*-values) was based on the suggestion of Wang et al. (2014a):

$$p=\frac{\text{y}1+\text{y}2}{n1+n2};q=1-p;\delta_{p1-p2}=\sqrt{pq\left(\frac{1}{n1}+\frac{1}{n2}\right)};U=\frac{p1-p2}{\delta_{p1-p2}}$$

where n1 represents the total number of fragments of a given sample, n2 the total fragments of another sample, y1 the total number of methylated fragments of a given sample values, y2 the total number of methylated fragments in another sample, p1 the proportion (%) of methylated fragments of a given sample values and p2 the proportion (%) of methylated fragments in another sample.

**Table 2:** **Sequences of adaptors and primers used for MSAP analysis**.

**Adaptors/primers**	**Sequence (5′–3′)**
*Eco*R I adaptor-1	CTCGTAGACTGCGTACC
*Eco*R I adaptor-2	AATTGGTACGCAGTCTAC
*Hpa* II/*Msp* I adaptor-1	GATCATGAGTCCTGCT
*Hpa* II/*Msp* I adaptor-2	CGAGCAGGACTCATGA
*Eco*R I pre-selective primer	GACTGCGTACCAATTCA
*Hpa* II/*Msp* I pre-selective primer	ATCATGAGTCCTGCTCGG
*Eco*R I selective primer-2	GACTGCGTACCAATTCAAG
*Eco*R I selective primer-3	GACTGCGTACCAATTCACA
*Eco*R I selective primer-4	GACTGCGTACCAATTCACT
*Eco*R I selective primer-5	GACTGCGTACCAATTCACC
*EcoR* I selective primer-6	GACTGCGTACCAATTCACG
*EcoR* I selective primer-7	GACTGCGTACCAATTCAGC
*Eco*R I selective primer-8	GACTGCGTACCAATTCAGG
*Hpa* II/*Msp* I selective primer-1	ATCATGAGTCCTGCTCGGTAA
*Hpa* II/*Msp* I selective primer-2	ATCATGAGTCCTGCTCGGTCC
*Hpa* II/*Msp* I selective primer-3	ATCATGAGTCCTGCTCGGTTC
*Hpa* II/*Msp* I selective primer-6	ATCATGAGTCCTGCTCGGTAG
*Hpa* II/*Msp* I selective primer-7	ATCATGAGTCCTGCTCGGTTG
*Hpa* II/*Msp* I selective primer-8	ATCATGAGTCCTGCTCGGTCA

## Results

### Pollen germination and callus induction from ovules

**Figure 1:**
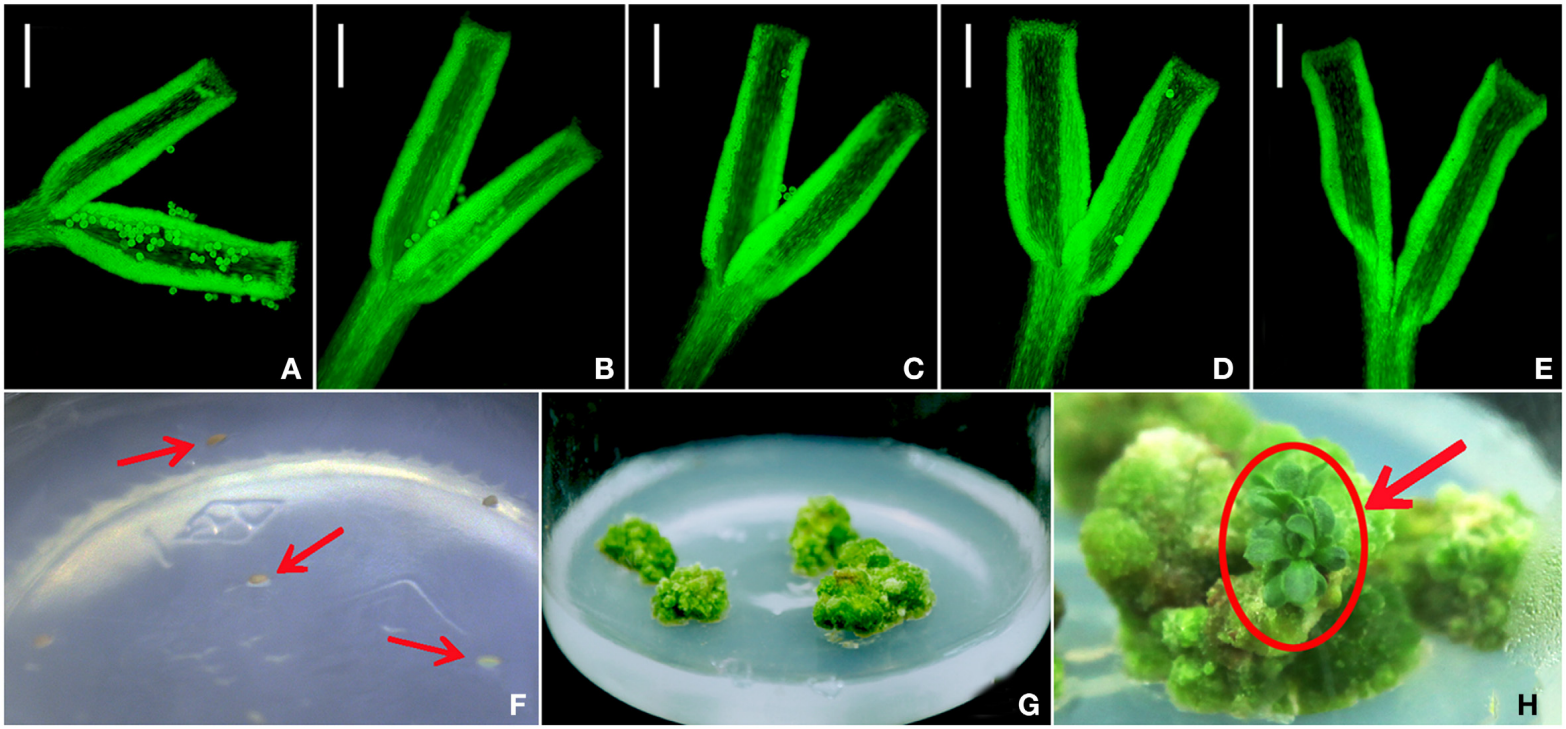
**The germination of *A. frutescens* pollen on the *C. morifolium* stigma imaged at (A) 6 h, (B) 12 h, (C) 24 h, (D) 36 h, and (E) 48 h post pollination and callus development from non-fertilized, *in vitro* cultured *C. morifolium* ovules. (F)** ovules, **(G)** formation of callus, **(H)** formation of plantlets. Bar **(A–E)**: 100 μm.

No pollen tube formation was detected over the period from 6 to 48 h post pollination (Figures 1A–E). From 2579 ovules only 105 produced any callus after 20 days in culture (Figure 1F). The calli became green, had a rough surface and were variable in size (Figure 1G), but only three of them went on to develop a somatic embryo: the first after 62 days in culture, the second after 68 days and the final one after 133 days (Figure 1H).

### Ploidy and origin of the regenerated plants and derived doubled haploids

**Figure 2:**
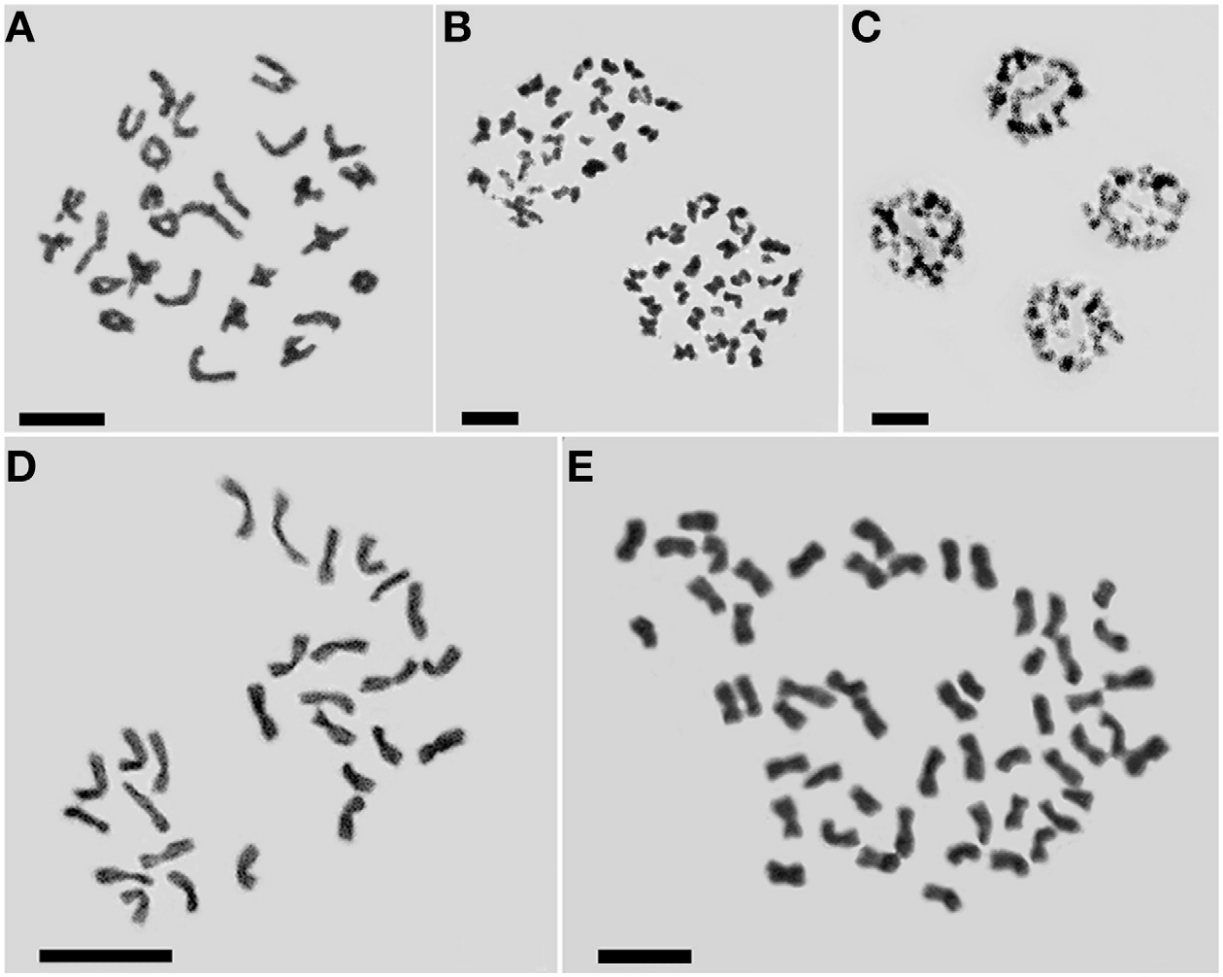
**Cytological characterization of *C. morifolium* cv. “Zhongshanzigui” and its haploid and doubled haploid derivatives**. **(A–C)** Meiotic chromosomes in the cv. “Zhongshanzigui” pollen mother cell: **(A)** metaphase I, **(B)** anaphase I, **(C)** telophase II. Somatic chromosomes in **(D)** haploid and **(E)** doubled haploid plants. Bar: 5 μm.

Meiotic analysis of “Zhongshanzigui” confirmed it as a diploidized allohexaploid (Figures 2A–C), with the expected chromosome number of 2*n* = 6*x* = 54. The three regenerated plantlets all developed into adult plants and reached flowering. On the basis of their somatic chromosome number, only one was a true haploid (2*n* = 3*x* = 27) (Figure 2D), whereas the other two were both hexaploid (2*n* = 6*x* = 54). The true haploid regenerated much later than the other two, and was also the slowest growing of the three regenerants.

**Figure 3:**
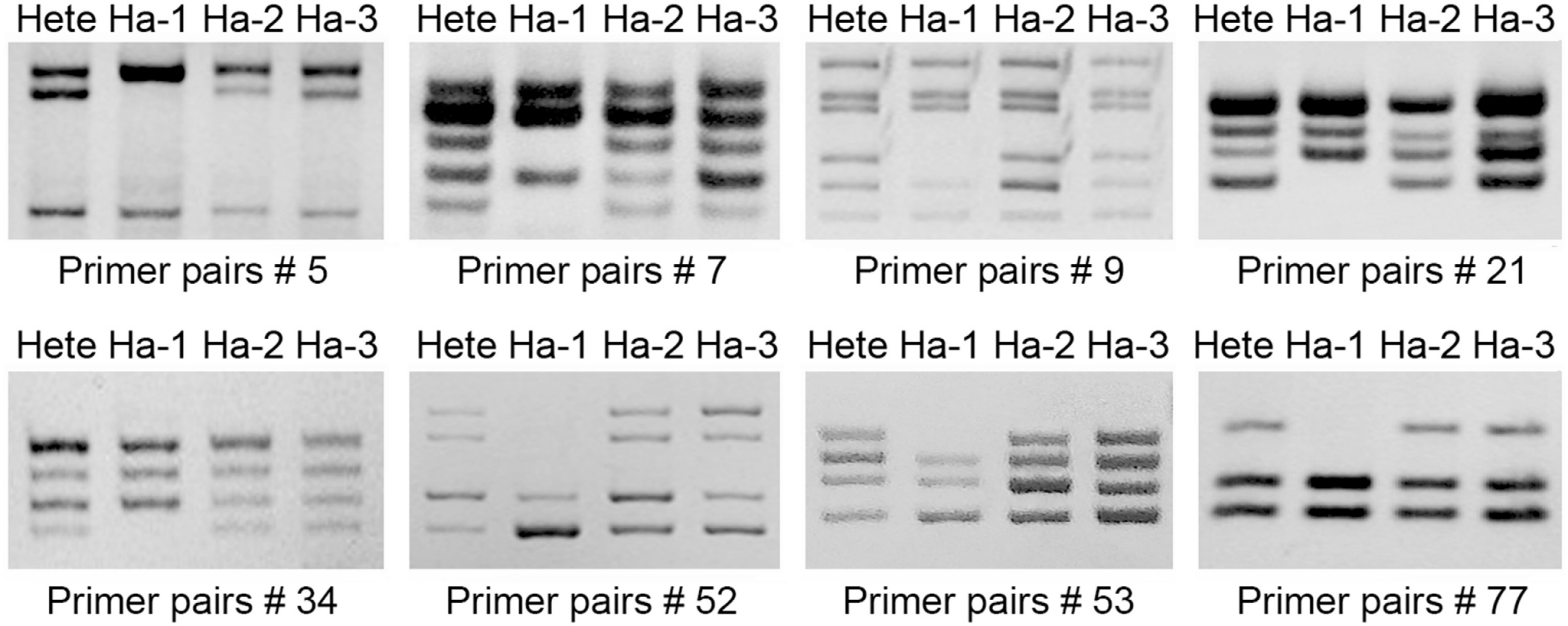
**Microsatellite fingerprinting reveals the genomic constitution of the regenerants from ovule culture**. Hete is cv. “Zhongshanzigui,” Ha-1 is a true haploid; Ha-2 and Ha-3 originate from somatic maternal tissue.

Microsatellite profiling of the three regenerants indicated that two hexaploid ones score the same microsatellite fragments, while the true haploid has fewer ones (Figure 3). The implication was each was derived from a somatic (rather than from a gametic) cell of the mother plant, and represented the product of a spontaneous whole genome doubling during the *in vitro* culture process. Following colchicine treatment, nine doubled haploid plants (Figure 2E), each bearing the expected chromosome complement, were derived from the cultured nodal segments of the confirmed haploid.

### Morphology of the haploid and derived doubled haploid plants

**Figure 4:**
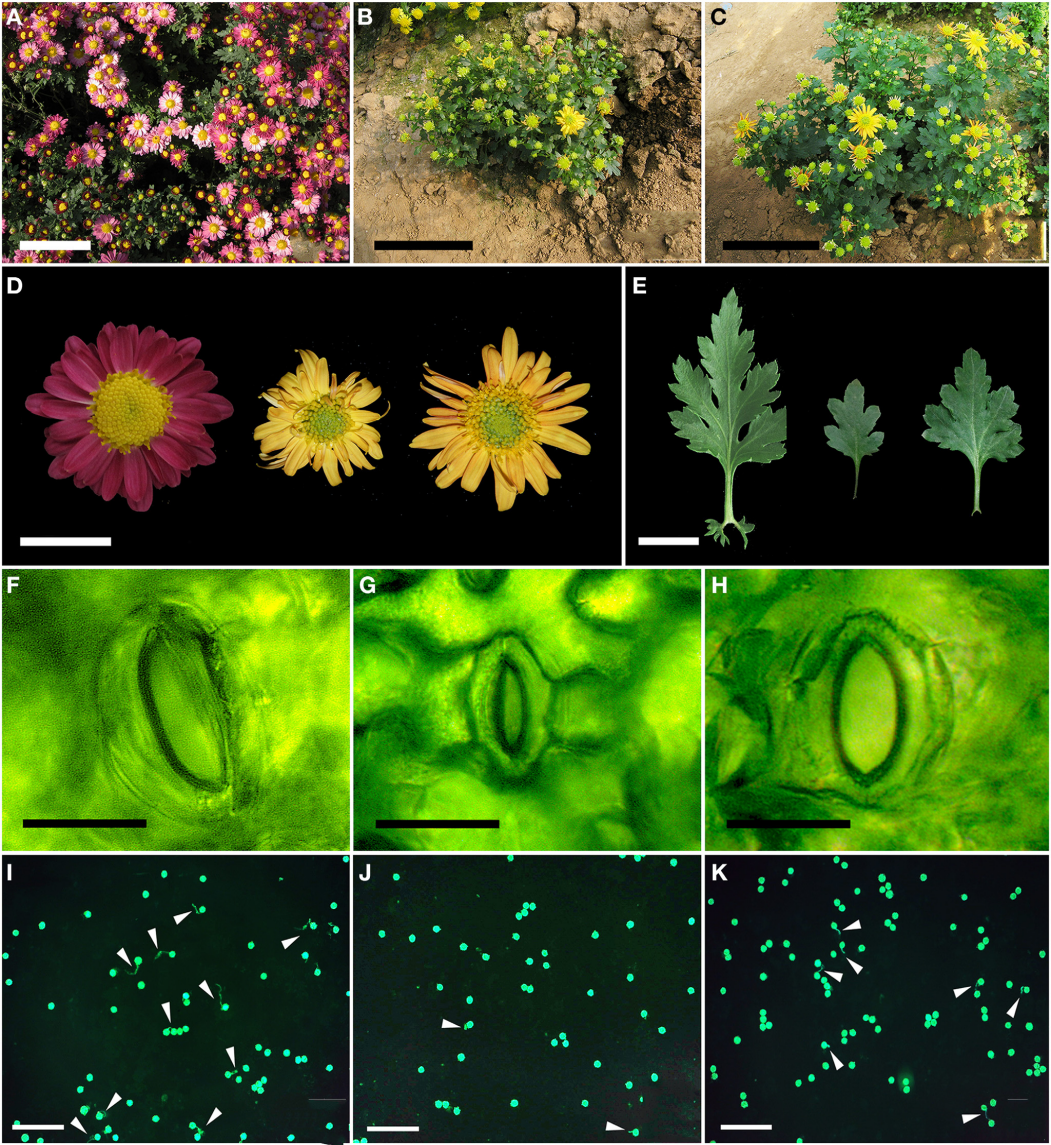
**Whole plant morphology of (A) cv. “Zhongshanzigui,” (B) the haploid, and (C) the doubled haploid plants**. Bar: 10 cm. **(D)** Leaf morphology of cv. “Zhongshanzigui” (left), the haploid (center) and the doubled haploid (right) plants. Bar: 2 cm. **(E)** Flower morphology of cv. “Zhongshanzigui” (left), the haploid (center) and the doubled haploid (right) plants. Bar: 2 cm. **(F–H)** Stomatal morphology of **(F)** cv. “Zhongshanzigui,” **(G)** the haploid and **(H)** doubled haploid plants. Bar: 30 μm. **(I–K)** Pollen viability of **(I)** cv. “Zhongshanzigui,” **(G)** the haploid and **(K)** doubled haploid plants. Arrows point to the pollen tubes. Bar: 200 μm.

Both the mother and the doubled haploid plants grew more vigorously than did the haploid plant, and formed larger plants at the adult stage (Figure 4, Table 3). The mean plant height of “Zhongshanzigui” was 89 cm, almost three times that of the haploid. The colchicine-doubled plants were taller than their haploid progenitor, but still only grew to half the height of “Zhongshanzigui.” Similarly, the leaf length, leaf width and flower diameter of “Zhongshanzigui” plants were all larger than their equivalents in the doubled haploids, which were in turn larger than those of the haploid (Figures 4A–E). The “Zhongshanzigui” inflorescence formed 177 tubular florets, compared to 96 in the haploid and 151 in the doubled haploids. Ligulate flower number was less variable: the number borne by the haploid inflorescence was greater than that by the doubled haploid inflorescence, but smaller than that by the “Zhongshanzigui” inflorescence (Figure 4D, Table 3). Interestingly, the color of the “Zhongshanzigui” inflorescence was mauve, while that of the haploid and doubled haploids was yellow (Figure 4D). The haploid's stomatal length was shorter than those of either “Zhongshanzigui” or the doubled haploids (Figures 4F–H). *In vitro* cultured pollen began to germinate within 2 h (data not shown), and by 8 h 17.0% of the “Zhongshanzigui” pollen had germinated; the proportion in the doubled haploids was 9.7%, while in the haploid it was only 1.3%. The pollen tubes formed by the few germinating grains of the latter plant were twisted and short, unlike those of the doubled haploids (Figures 4I–K).

**Table 3:** **The morphology of “Zhongshanzigui” and its derived haploid and doubled haploid progeny**.

**Characters**	**Heterozygote**	**Haploid**	**Di-haploid**
Plant height (cm)	89.33 ± 3.98^a^	28.42 ± 2.25^c^	45.52 ± 2.45^b^
Leaf length (cm)	7.83 ± 0.31^a^	3.66 ± 0.25^c^	6.07 ± 0.36^b^
Leaf width (cm)	4.76 ± 0.23^a^	2.29 ± 0.20^c^	3.36 ± 0.17^b^
Ligulate flower quantity	44.30 ± 0.95^a^	33.40 ± 1.35^b^	27.70 ± 2.41^c^
Tubular florets quantity	176.80 ± 5.09^a^	96.00 ± 1.89^c^	151.00 ± 2.54^b^
Flower diameter (cm)	4.36 ± 0.33^a^	3.15 ± 0.20^c^	3.96 ± 0.35^b^
Stomata length (μm)	43.71 ± 1.35^a^	33.60 ± 1.56^c^	38.93 ± 1.40^b^
Pollen viability (%)	17.01% ± 1.11^a^	1.27% ± 0.35^c^	9.69% ± 0.10^b^

*Values (mean ± SE) associated with a different superscript (a, b, c) differ significantly from one another (p < 0.01)*.

### Changes of cytosine methylation in the haploid and doubled haploid plants

**Figure 5:**
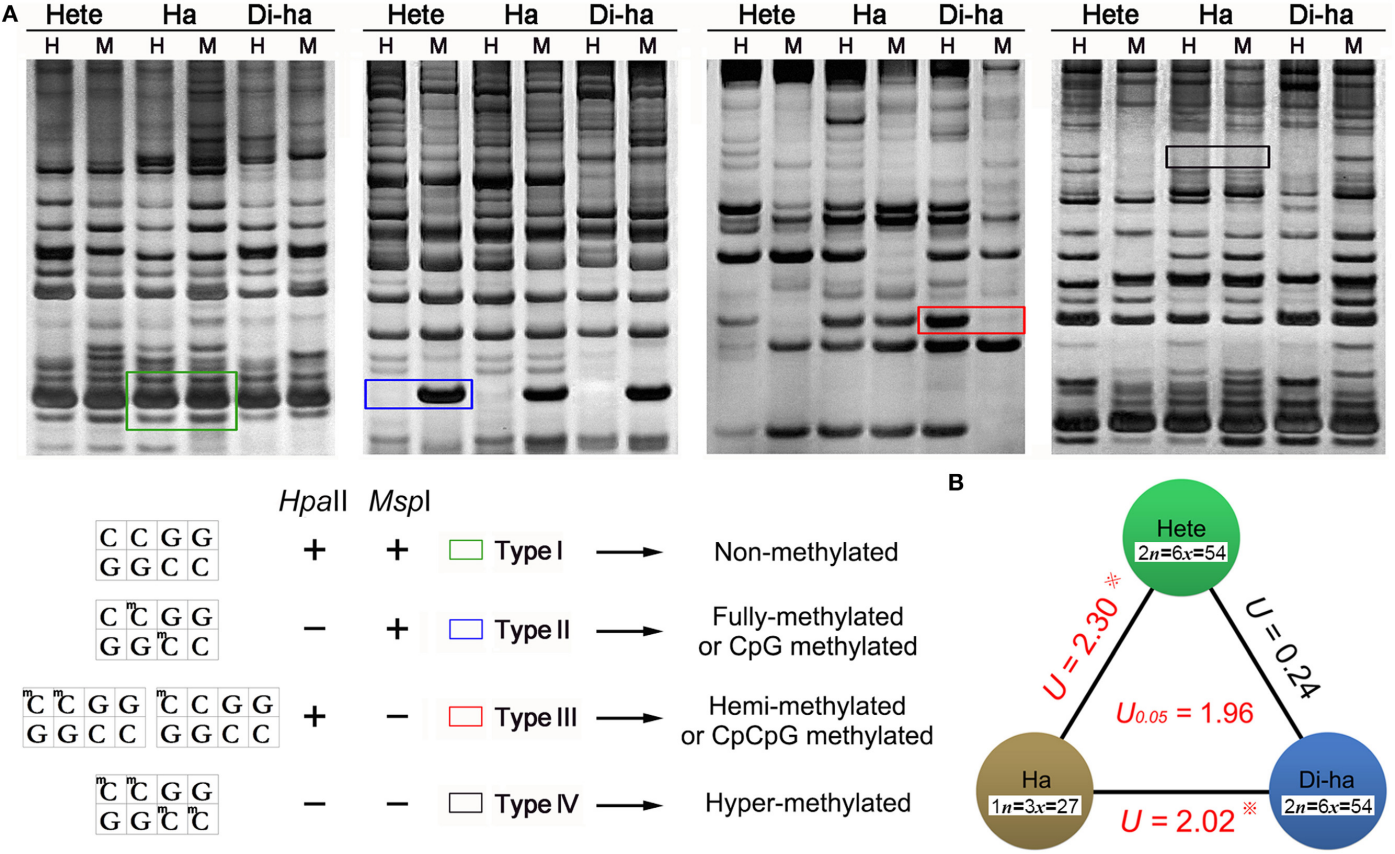
**Representative fragments in MSAP profiles. (A)** *Hpa* II and *Msp* I sensitivities to 5-CCGG methylation status (“+”/“−”: enzyme can/cannot cut): The four types of fragment generated. Type I: non-methylated, appearing in both the H and M lanes; Type II: fully methylated, present in the M but not the H lanes; Type III: hemi-methylated, present in the H but not the M lanes; Type IV: hyper-methylated, not present in either the H or M lanes. **(B)** The *U*-values within the three forms of chrysanthemum. A higher *U*-value implies a larger methylation difference between different samples, but only *U*-value > 1.96 was statistically significant. Hete: cv. “Zhongshanzigui”; Ha: haploid; Di-ha: doubled haploid.

MSAP profiling was based on an isoschizomeric pair of restrictions enzymes, where one cleaves irrespective of the methylation status at the recognition sequence, while the other only cleaves when the recognition site is unmethylated. The enzyme pair *Msp* I and *Hpa* II are particularly suitable, as their recognition sequence (5′-CCGG-3′) includes a CpG dinucleotide. The latter does not cleave if either cytosine is fully methylated, but will do so if the external cytosine is hemi-methylated (CpCpG methylated, only one DNA strand methylated); in contrast, *Msp* I will not cleave if the external cytosine is fully- or hemi-methylated; the both eazymes do not cleave if the both cytosines fully methylated (McClelland et al., 1994; Sha et al., 2005). Therefore MSAP based on *Hpa* II and *Msp* I cleavage can discriminate four kinds of methylation state: Type I is non-methylated site, Type II is fully-methylated or CpG methylated site; Type III is hemi-methylated or CpCpG methylated site; Type IV is hyper-methylated site (Figure 5A).

Applying the full set of 30 MSAP primer combinations identified 1008 fragments. Of these, 52.2% were methylated in the “Zhongshanzigui” genome, 47.0% in the haploid's and 51.7% in the doubled haploids'. A higher *U*-value implies a larger difference between different samples, but only *U*-value > 1.96 [*U*_(0.05)_ = 1.96] was statistically significant. The *U*-value of the haploid with respect to “Zhongshanzigui” was 2.30, while it was 2.02 between the haploid and the doubled haploids. The difference between “Zhongshanzigui” and the doubled haploids was statistically insignificant (*U* = 0.24) (Figure 5B). It should be noted that each pair of biological replicates manifested a near-complete uniformity in MSAP profile, as did all nine of the doubled haploids (Supplementary Figure 1); thus, chromosome doubling might be a highly conservative process with respect to cytosine methylation.

Specifically, most, but not all of the MSAP fragments in the “Zhongshanzigui” profile were shared by the haploid's and the doubled haploids'. As a result of haploidization of cv. “Zhongshanzigui,” of the 94 fragments which varied for cytosine methylation status between the haploid and the maternal cultivar, 35 (37%) were methylated in the former: 26 involved hemi-methylation, eight full methylation and one hyper-methylation. The other 59 (63%) fragments were demethylated in the haploid, 47 switching from hemi-methylation, nine from full methylation and three from hyper-methylation. Following the chromosome doubling of the haploid, of these, 70 (73%) became methylated in the doubled haploids (32 changed from hemi-methylated and 20 from non-methylated), while 18 moved from non-methylated to hyper-methylated. The chromosome doubling induced de-methylation in 26 fragments, of which seven changed from hyper-methylated to non-methylated and 19 sites from hemi- or fully-methylated to non-methylated. As a whole, the frequency of demethylation was 1.69-fold that of methylation as induced by haploidization. While from haploid to double haploid, the frequency of demethylation was only 0.38-fold of the frequency of methylation.

## Discussion

### Induction of haploidy in *C. morifolium*

Attempts to induce chrysanthemum haploids from either immature anthers or microspores have been largely unsuccessful to date; although regenerants have been reported, their somatic chromosome number proved to be the same as that of the progenitor plant (Yang et al., 2005). Prior unpublished experiments conducted in our laboratory have similarly failed to yield haploids, whether the protocol was based on either isolated microspores or non-stimulated ovules. In other species, both haploids and doubled haploids have been induced from non-fertilized ovule explants (Chen et al., 2011). The pre-treatment of the explant by germinating incompatible pollen on the stigma, as implemented in the current study, can be an effective means of increasing the likelihood of recovering haploid plants, as shown for a range of Japanese *Chrysanthemum* species (Watanabe, 1977; Wędzony et al., 2009), saffron, sugar beet and carnation (Gürel et al., 2000; Sato et al., 2000; Sulistyaningsih et al., 2006). Nevertheless, from a total of over 2500 cultured, pre-treated ovules, only three plants were successfully regenerated, and of these just one was a true haploid, whereas the remaining two regenerants were both allohexaploid and should be derived from heterozygous somatic cell (Figure 3). Clearly, improvements will need to be made to the haploidization protocol before haploid recovery can be considered routine in chrysanthemum.

Where spontaneous chromosome doubling is rare, colchicine treatment can provide a ready means for its induction (Li et al., 2013a). Cytogenetic analysis has shown that the present results are in good agreement with previous studies on the colchicine treatment of plants or plantlets (Chen et al., 2011; Li et al., 2013b). Apart from the direct identification of ploidy obtained via karyotypic analysis, plant morphology, in the form of plant height, leaf size, flower diameter and stomatal length were all usable here to distinguish between haploid and doubled haploid plants. The haploid grew less vigorously than the mother cultivar; its only advantage over the diploid form related to the number of ligulate flowers developed in the inflorescence. Surprisingly, the color of both the haploid's and the doubled haploids' inflorescence was yellow rather than mauve, which may be due to expressions of some recessive genes gene(s) involved in regulating flower color.

### Variation of cytosine methylation in heterozygote, haploid and Di-haploid

The MSAP profiling showed that the haploid experienced a significantly lower frequency of cytosine methylation than did either the maternal diploid (*U* = 2.30) or the doubled haploid (*U* = 2.02), but that there was no significant difference in frequency between the diploid and the doubled haploids (*U* = 0.24). It is possible, of course, that some variation in heritable cytosine methylation may have been induced during the callus phase (Miguel and Marum, 2011), but it is impossible to precisely quantify this source of variation. For this reason, it is difficult to precisely quantify the severity of this change caused by the tissue culture step. However, it reported that changes in cytosine methylation caused by tissue culture are thought to be rare in *Codonopsis lanceolata* (Guo et al., 2007), while in oil palm, the frequency of this event is low (~0.3%) (Matthes et al., 2001). In rice, such changes have been shown to be concentrated within a relatively small proportion of the genome using whole genome bisulfite sequencing (Stroud et al., 2013). All these studies indicated that haploidization rather than the tissue culture step may play the major role in altering methylation in these chrysanthemum lines.

The influence of polyploidization on epigenetic status has been studied in some detail in the allotetraploid synthesized from the interspecific hybrid *Arabidopsis thaliana x A. lyratara*. In this system, Beaulieu et al. (2009) used an MSAP platform to show that around 25% of MSAP fragments suffered a change in their cytosine methylation status, frequency which contrasts to the figure of just ~9% figure which applied to the comparison between “Zhongshanzigui” and its haploid (or doubled haploid) derivatives. It is recognized that any potential CpG sites present in the heterozygous state in “Zhongshanzigui” may not have been inherited by the haploid following segregation, thus producing an over-estimate of the actual number of de-methylation events; however, even so the frequency of de-methylation was still 1.7 times that of methylation. The implication is that haploidization favors de-methylation. Some adjustments in DNA methylation were clearly induced by the chromosome doubling process, but here it was methylation which was favored. The MSAP data implied that globally, methylation was about 2.7-fold more frequent than de-methylation.

Hyper-methylation tends to be associated with gene silencing, and hypo-methylation with gene activation (Martienssen and Colot, 2001; Wang et al., 2006). Cytosine methylation has been documented as having a marked effect on cell differentiation, chromatin inactivation and genomic imprinting and is known to be affected by chromosome doubling (Finnegan, 2001, 2002; Liu and Wendel, 2003). As an example, among F_1_ hybrids between diploid species in the genera *Aegilops* and *Triticum* group, an estimated 13% of genes suffered cytosine methylation as a result of genomic shock (Shaked et al., 2001), a 2-fold difference was observed in the level of cytosine methylation between reciprocal F_1_ interspecific *Cucumis* spp. hybrids and their allotetraploid, with 68% of sites becoming cytosine methylated and 32% becoming de-methylated in the allotetraploid (Chen et al., 2007). Here, about 9% of the sites were variable, and of these, 37% became methylated as a result of haploidization and 73% as a result of whole genome duplication. The expectation was therefore that evidence would be found for alterations in cytosine methylation and have had a potential influence on the haploid and double haploid chrysanthemum plants.

Notably, in angiosperm female gametogenesis only half of the endogenous sequences or genes present in the heterozygotes will be present in the haploids. The difference in chromosomal constitution between parental cultivar and haploids might have an effect on gene expression, cell differentiation, chromatin inactivation and genomic imprinting via positive or passive demethylation of their DNA (Saze et al., 2003; Henderson and Jacobsen, 2007). This may be the reason the haploid displayed a high *U*-value (2.30) compared with cv. “Zhongshanzigui” (Figure 5B). Moreover, the original epigenetic makeup of the cultivar was restored in our hands in the doubled haploid via whole-genome duplication with no significant (*U* = 0.24) between cv. “Zhongshanzigui” and doubled haploids (Figure 5B). It could therefore be hypothesized that this alteration may secure appropriate maintenance as well as flexibility of epigenetic determination within three forms of chrysanthemum. Even though there was no significant difference (52.2 vs. 51.7%) between the frequency of methylated MSAP fragments in “Zhongshanzigui” and the doubled haploids, their detailed pattern of methylation was different, accounting perhaps in part for the differences in phenotype between them.

## In conclusion

The present study represents the first report of the production of haploid and doubled haploid chrysanthemum plants derived from the *in vitro* culture of non-fertilized ovules, morphological traits were changed significantly in the haploid plants. MSAP profiling suggested that alterations in cytosine methylation occurred as a result of both the haploidization and the chromosome doubling processes.

## Author contributions

Conceived and designed the experiments: Haibin Wang, Fadi Chen, Jiafu Jiang, Sumei Chen. Performed the experiments: Haibin Wang, Fadi Chen, Jiafu Jiang. Analyzed the data: Haibin Wang, Bin Dong. Contributed reagents/materials/analysis tools: Weimin Fang, Zhiyong Guan. Wrote the paper: Haibin Wang, Jiafu Jiang, Sumei Chen. All authors read and approved the final manuscript.

### Conflict of interest statement

The authors declare that the research was conducted in the absence of any commercial or financial relationships that could be construed as a potential conflict of interest.

## Supplementary material

Supplementary Figure 1 **Examples of MSAP profiles implying cytosine methylation alterations between biological replicates**. The *Hpa*II lanes involve the primer combination E4+HM7, and the *Msp*I lanes E8+HM8. Hete: “Zhongshanzigui”; Ha: haploid; Di-ha: doubled haploid. Two individual plants from each line (one marked with an asterisk, the other not) were selected at random.
