# Supplementary figures and images for: Characterization of in vitro haploid and doubled haploid Chrysanthemum morifolium plants via unfertilized ovule culture for phenotypical traits and DNA methylation pattern
Source: Front Plant Sci. 2014 Dec 22;5:738. doi: 10.3389/fpls.2014.00738 (PMC4273617; doi:10.3389/fpls.2014.00738)

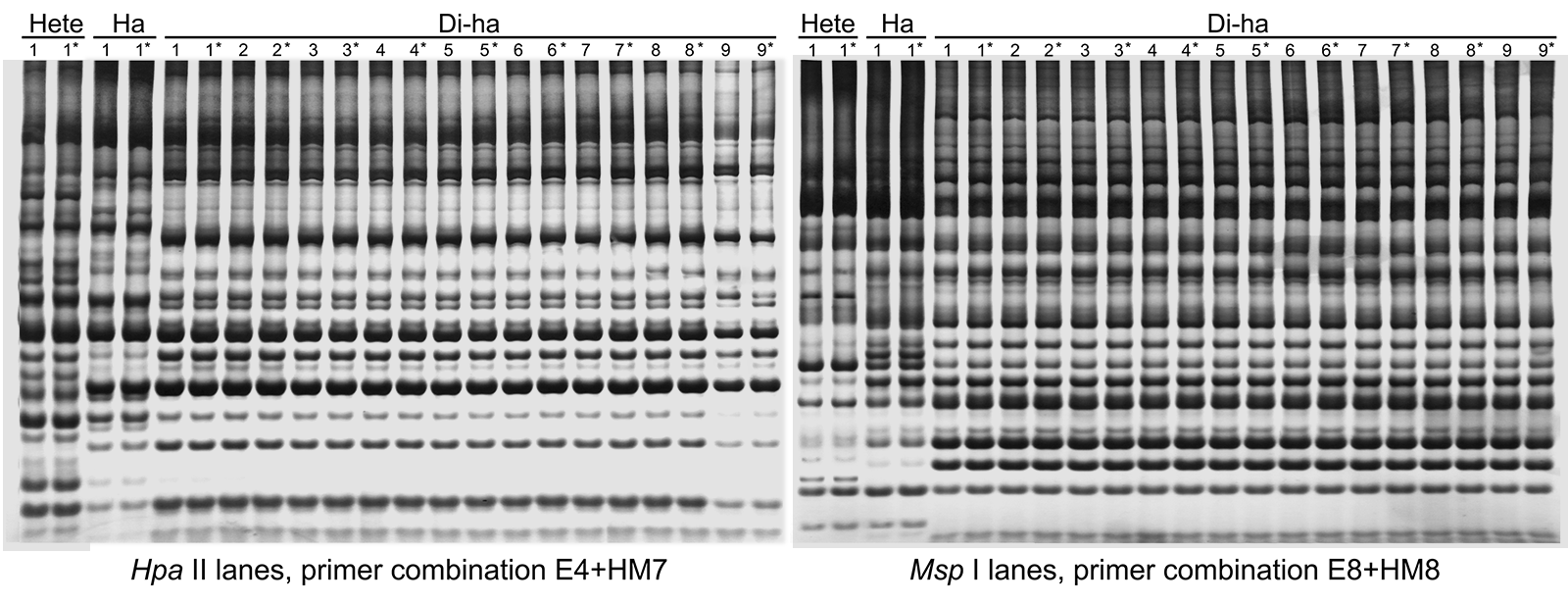

Supplement: Supplementary Figure 1 — Examples of MSAP profiles implying cytosine methylation alterations between biological replicates. The HpaII lanes involve the primer combination E4+HM7, and the MspI lanes E8+HM8. Hete: “Zhongshanzigui”; Ha: haploid; Di-ha: doubled haploid. Two individual plants from each line (one marked with an asterisk, the other not) were selected at random. [file Image1.TIF]
